# A Personalized Energy Expenditure Estimation Method Using Modified MET and Heart Rate-Based DQN

**DOI:** 10.3390/s25113416

**Published:** 2025-05-29

**Authors:** Min-Seo Kim, Ju-Hyeon Seong

**Affiliations:** Department of Maritime AI and Cyber Security & Interdisciplinary Major of Maritime AI Convergence, National Republic of Korea Maritime & Ocean University, 727, Taejong-ro, Yeongdo-gu, Busan 49112, Republic of Korea; sea7616@g.kmou.ac.kr

**Keywords:** energy expenditure estimation (EEE), wearable device, MET, reinforcement learning (RL)

## Abstract

Wearable device-based personal activity measurement technology provides various personalized services by integrating bio-signals. However, accurately and rapidly estimating energy expenditure (EE) remains challenging due to user movement and the limitations of measurement parameters. In this paper, we propose Real-Time Energy Expenditure (RTEE), a novel real-time and personalized energy expenditure estimation (EEE) method. The proposed RTEE integrates a Deep Q-Network (DQN)-based activity intensity coefficient inference network with a modified energy consumption prediction algorithm to estimate energy expenditure based on real-time variations in the user’s heart rate measurements. Therefore, the proposed algorithm can be applied to various heart rate-based energy consumption prediction methods.

## 1. Introduction

Recently, research on the accuracy of bio-signal measurement based on wearable devices has been actively conducted [1,2]. Wearable devices, such as smart rings and watches, measure bio-signals in real-time, including heart rate, oxygen saturation, and skin temperature. Based on the measured bio-signals, wearable devices can determine information such as personal activity status and health status [3]. Among these, energy expenditure (EE), which provides information to determine the user’s level of activity, calculates activity levels by distinguishing body movements, making it possible to determine. So, it is possible to determine whether the user is fatigued or overworked. Moreover, by integrating biomechanical motion patterns with physiological data, it is possible to infer the specific activities being performed by the user in real-time [4]. Personalized physiological and activity data play a crucial role in real-time health monitoring, risk assessment, and emergency response, significantly contributing to individual healthcare [5].

Among wearable devices, smartwatches are the most widely adopted due to their high usability and convenience. When using wearable devices to assess the energy expenditure of workers, it is essential to ensure that the device does not pose a safety risk or interfere with work tasks. Smart rings may interfere with precise hand movements required for certain occupations and pose a potential safety risk due to their conductive metal exterior, which can conduct electricity. Therefore, for monitoring workers’ activity levels, smartwatches are more suitable, as they do not hinder finger movements and provide insulation through rubber wristbands. Smartwatches are equipped with photoplethysmography (PPG) sensors, enabling real-time measurement of heart rate (HR), heart rate variability (HRV), and oxygen saturation (SpO_2_). Since heart rate is strongly correlated with exercise intensity, it can be used to estimate exercise intensity and infer EE.

Various methods exist for estimating EE, including the oxygen consumption (VO_2_) method, metabolic equivalent of task (MET) method, and heart rate-based method. The VO_2_ method, derived from indirect calorimetry, calculates EE by measuring the amount of oxygen consumed by the body. This method directly reflects metabolic activity and is considered the most accurate approach for estimating EE [6,7,8]. However, the VO_2_-based method requires a sensor to be attached to the mouth, limiting its practical usability.

The MET method quantifies EE by comparing it to the resting metabolic rate (RMR) [9,10,11]. This approach has the benefit of enabling simple EE estimation using only MET values for each activity type, body weight, and exercise duration. Thus, it is widely used in wearable devices such as smartwatches for daily EE measurement [12]. However, the MET method has limitations as it does not account for changes in EE based on the exercise environment (e.g., outdoor vs. indoor), even within the same type of activity. Additionally, accurately applying MET indices requires information on both the user’s current activity type and exercise intensity. However, real-time identification of activity types and intensities is challenging. To address this, researchers have proposed methods that use cameras to recognize user activities [13,14]. Nevertheless, these methods face practical limitations, including difficulty in accurately recognizing movements and the need for the camera to track the user during activities like running. Furthermore, cameras alone cannot effectively assess exercise intensity variations caused by differences in movement speed.

EE can be estimated using the Keytel equation, which is based on heart rate [15,16,17]. This approach has the advantage of reflecting exercise intensity in real-time through variations in heart rate, allowing for dynamic and individualized EE estimation [18,19]. However, the Keytel equation assumes a proportional relationship between heart rate and VO_2_, which may lead to inaccuracies as the proportionality factor varies across different exercise intensities, reducing its precision [20,21].

To overcome these limitations, we propose a novel real-time and personalized EE estimation method, referred to as RTEE. The proposed method integrates conventional EE estimation formulas with a deep Q-network (DQN)-based exercise intensity inference model to predict EE based on real-time heart rate data. To improve real-time EE accuracy, a DQN-based, heart rate-driven exercise intensity prediction network is employed. Consequently, the proposed RTEE provides fine-grained, real-time EE estimation per second by leveraging heart rate variations that reflect an individual’s health and activity status.

## 2. Related Works

### 2.1. Energy Expenditure Estimation

There are three main methods for energy expenditure estimation (EEE): indirect calorimetry, MET-based estimation, and heart rate-based estimation. Indirect calorimetry estimates energy expenditure (EE) by measuring oxygen consumption. The formula for calculating EE per minute is as follows:

(1) $$EE(kcal/min)=\frac{{VO}_{2}\times Weight\times 5}{1000}$$

Here, VO_2_ [mL/kg/min] represents the amount of oxygen consumed per minute per kilogram of body weight, and Weight (kg) refers to the user’s body weight. Indirect calorimetry is considered the most accurate method to estimate EE because it calculates metabolic activity with reference to VO_2_ [6,7,8]. However, its use in daily life is limited, as it requires wearing a VO_2_ sensor attached to the mouth to measure oxygen consumption and obtain the VO_2_ value.

The second method, the MET-based approach, quantifies the relative intensity of an activity by comparing energy expenditure (EE) with the resting metabolic rate (RMR). This method enables the calculation of EE for different activity types [9,10,11]. The resting state is defined as 1 MET, and activity intensity is quantified relative to this baseline. The formula for estimating EE using MET is as follows:

(2) $$EE(kcal/min)=\frac{MET\times Weight\times 3.5}{200}$$

where MET represents the activity index based on activity type. Representative MET values for various activities are shown in Table 1.

Table 1 shows the representative MET index values for various activity types. A MET index of 1, representing a baseline state, corresponds to sitting or lying down, where minimal energy is expended without movement, indicating a resting state. For sedentary activities such as computer work, document processing, and meetings, the MET index is defined as 1.5, representing low energy expenditure. In contrast, during exercise activities such as cycling, the MET index varies depending on speed. For instance, cycling at 10 mph corresponds to 4 MET, whereas speeds of 20 mph or higher are classified as 16 MET. Additionally, for physical labor, activities such as food serving are assigned a MET index of 8, while firefighting work is classified as 13 MET.

If RMR and EE are known, the MET value can be easily calculated using the following equation [10]:

(3) $$MET=\frac{EE}{RMR}$$

Although MET-based EE estimation is a simple method, it does not account for variations in energy expenditure (EE) due to individual physiological differences, environmental conditions (e.g., terrain slope, indoor vs. outdoor activities), or changes in movement intensity. Furthermore, EE estimation is only feasible when the activity type is accurately identified beforehand, making real-time application challenging.

The third approach for EE estimation is the heart rate-based method [15,16,17]. This method has the advantage of incorporating exercise intensity into EE estimation by leveraging the relationship between heart rate and exercise intensity [18,19]. Equation (4) represents the Keytel equation for EE estimation:

(4) $$EE\left(kJ/min\right)=Gender\times (-55.0969+0.6309\times {HR}_{act}+0.1988\times Weight+\;0.2017\times Age+\left(1-Gender\right)\times (-20.4022+0.4472\times {HR}_{act}-\;0.1263\times Weight+0.074\times Age)$$

Here, *Gender* is defined as 1 for males and 0 for females, and ${HR}_{act}$ represents the current heart rate. The Keytel equation is based on the assumed proportional relationship between heart rate and VO_2_. However, in practice, this proportionality varies across different exercise intensities, potentially leading to estimation errors depending on exercise conditions [20,21].

The MET-based and Keytel EE estimation methods are currently the most widely used approaches for EEE. Although subsequent research has introduced more precise EE estimation models, many of these require additional equipment or data that are difficult to obtain in daily life. Consequently, the MET-based and heart rate-based methods remain the most practical for applications [22,23,24].

This study proposes a novel EEE method that predicts exercise intensity in real-time based on heart rate data. The proposed method is based on the principle that exercise intensity can be inferred from heart rate and, similar to the MET-based method, incorporates exercise intensity into the resting metabolic rate (RMR) to estimate energy expenditure (EE). This study aims to develop an EEE approach that can be implemented in smartwatches using minimal input data, ensuring feasibility for everyday use. Accordingly, the proposed method is compared with two widely used approaches to evaluate its effectiveness.

### 2.2. Resting Metabolic Rate

EE is calculated as the sum of resting metabolic rate RMR and activity energy expenditure AEE, where RMR refers to the energy required to sustain vital bodily functions and AEE corresponds to the additional energy consumed during physical activity [25,26]. This relationship is expressed as:

(5) EE=RMR+AEE

RMR is commonly estimated using the Mifflin-St Jeor equation [27,28], which is also employed in this study. This equation estimates daily RMR based on basic personal attributes such as gender, weight, height, and age, as shown in Equations (6) and (7):

(6) $${RMR}_{day\;}(Male)=\left(10\times Weight\right)+\left(6.25\times Height\right)-\left(5\times age\right)+5$$

(7) $${RMR}_{day\;}\left(Female\right)=\left(10\times Weight\right)+\left(6.25\times Height\right)-\left(5\times age\right)-161$$

Since these equations provide daily RMR values, they are divided by time to obtain RMR per minute or per second.

The Mifflin-St Jeor equation was originally developed to estimate basal metabolic rate (BMR); however, it has been widely used in research and practical applications for estimating resting metabolic rate (RMR) as well [29]. The numerical difference between BMR and RMR can vary depending on individual physiological conditions, and since the Mifflin formula is a predictive approximation, it inherently involves estimation error. Consequently, many studies do not strictly distinguish between the two concepts and use them interchangeably. Although BMR and RMR differ in their definitions and measurement conditions, previous studies have reported that they may differ by approximately 10% in value [30].

### 2.3. Reinforcement Learning

Reinforcement learning (RL) is a machine learning approach in which an agent interacts with an environment to learn an optimal policy that maximizes rewards. In supervised learning, a model is trained using ground truth (GT) data, whereas in RL, the agent learns the policy through trial and error, aiming to maximize rewards without the need for GT data.

Deep Q-Network (DQN) is an RL approach that combines convolutional neural networks (CNNs) with traditional Q-learning, where the Q-function is approximated using a CNN [31,32]. DQN has been successfully applied to environments with continuous or complex state and action spaces, overcoming the limitations of conventional Q-learning. As a result, it is now widely utilized across various domains [33,34].

Due to the intrinsic characteristics of RL, which infers values without explicit labels, it is particularly suitable for applications in biomedical fields, where precise measurements are challenging and data collection is complex. RL has been extensively adopted in healthcare and bio-signal analysis. Recent studies have applied RL to develop behavior optimization policies for improving cycling posture based on sensor data collected during cycling sessions [35]. Additionally, RL algorithms have been introduced to optimize deep brain stimulation (DBS) for Parkinson’s disease treatment, enabling the algorithm to learn DBS patterns and identify the optimal stimulation pattern, thereby enhancing therapeutic efficacy [36]. In addition, research is being conducted on applying RL to wearable devices. Research has been conducted on applying RL to power management of wearable devices [37]. In this study, we leverage the advantages of DQN to develop a model for predicting EE by integrating reinforcement learning with physiological modeling-based EE estimation formulas.

## 3. Methods

### 3.1. Overview of the Proposed Method

In this study, Deep Q-Network (DQN) was selected as the reinforcement learning model, considering the characteristics of the task, which involves real-time energy expenditure prediction based on heart rate. DQN offers advantages in terms of stable convergence during training and ease of implementation. It is particularly well-suited for problems with discrete and relatively limited state and action spaces.

The action space in this study consists of 201 discrete activity intensity coefficients, divided into increments of 0.1. The reinforcement learning model is thus required to select the most appropriate value from these 201 candidates. This discrete action selection aligns naturally with the Q-value-based decision mechanism of DQN, making it a suitable choice for this problem setting.

Although alternative approaches such as actor-critic algorithms are widely used in reinforcement learning, they generally involve simultaneous optimization of policy and value functions, which increases computational complexity and introduces additional stability challenges [38,39]. In contrast, DQN focuses solely on value function approximation, resulting in lower computational overhead and more stable training dynamics. Given the real-time constraints of the application, computational efficiency and stability were critical considerations, leading to the selection of DQN as the most appropriate reinforcement learning framework for this study.

**Figure 1:**
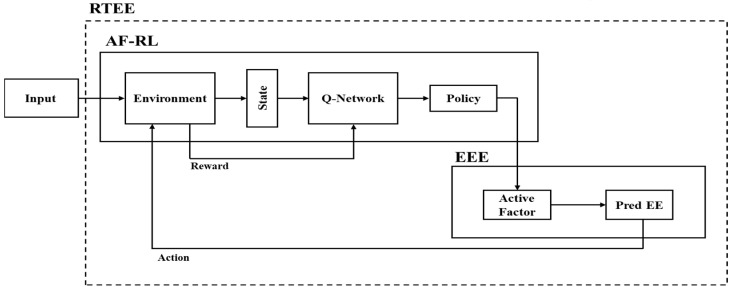
Overall schematic diagram of the proposed method.

The proposed RTEE algorithm estimates EE by combining real-time heart rate values, which are corrected using DQN, with a modified MET. Figure 1 shows the overall structure of RTEE, a novel energy consumption prediction method proposed. RTEE consists of the AF-RL part that predicts the activity intensity coefficient as RL and the EEE part that calculates the predicted EE. Data including personal body information and heart rate information for 300 s are input into AF-RL, and this data are input into the Environment.

In the Environment, 1 s of data are given as the State, and the Agent predicts the activity intensity coefficient a based on the user’s body information and heart rate information presented in the State. The EEE that receives the prediction calculates the real-time EE through modified MET using the personal information provided in the State. The calculated real-time EE is then sent back to the Environment to calculate the error with the ground truth (GT) EE. This process is repeated to predict the optimal activity intensity coefficient a. Therefore, real-time EE prediction is possible, and the structure can be personalized according to the individual’s health status.

**Figure 2:**
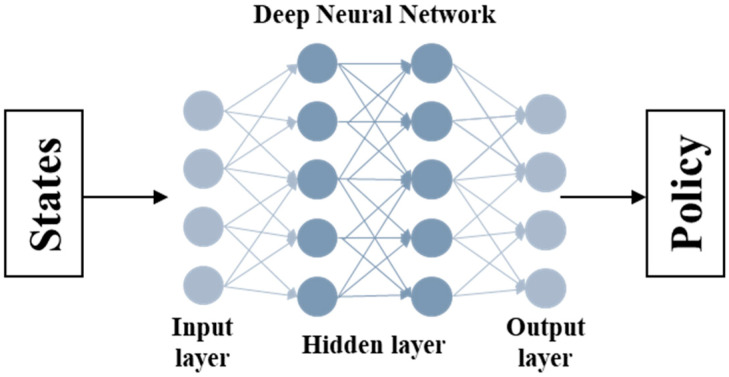
Overall Policy Network.

Figure 2 is a schematic diagram of the policy network. The policy network of the DQN model consists of a fully connected feedforward neural network. The input to the network includes five features: weight, height, age, gender, and heart rate. The network architecture comprises two hidden layers with 64 neurons each, followed by ReLU activation functions. The output layer consists of 201 units corresponding to discrete activity intensity coefficients, with a linear activation function.

### 3.2. Proposed AF-RL

**Figure 3:**
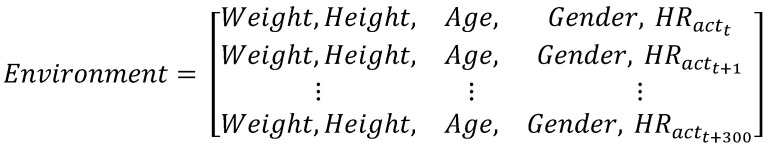
The structure of data input to the Environment of the AF-RL.

Proposed AF-RL, a DQN-based activity intensity factor (a) inference network, predicts real-time activity intensity based on heart rate. Figure 3 shows the input data provided as the model’s Environment. One Environment is defined as 5 min (300 s) of data consisting of [Weight, Height, Age, Gender, current heart rate] at 1 s intervals. The Environment has the entire 300 s data and provides the State to the Agent at 1 s intervals as follows.

(8) $$State=[Weight,Height,Age,Gender,{\;HR}_{{act}_{t}}]$$

The agent receives a State, defined as in Equation (9), and establishes a policy to estimate the optimal *activity* intensity coefficient. The action space available to the agent, based on the policy, is defined as follows:

(9) $$Action\left(A\right)=a,where\;a\in \{0,0.1,0.2,\ldots ,19.8,19.9,20\}$$

Here, a represents the activity intensity coefficient. The activity intensity coefficient ranges from 0 to 20 and is divided into 0.1-unit increments, resulting in a total of 201 possible values, from which one is selected as a. The selected activity intensity coefficient a is transferred to EEE and applied to calculate the predicted EE. The predicted EE, computed in EEE, is then transferred back to the Environment, where it is compared with the GT EE to calculate the error. The Agent receives a reward based on this comparison. The reward function is as follows:

(10) $$Reward=\frac{-\left|{GT}_{EE}-\right.\left.{Pred}_{EE}\right|}{1+{GT}_{EE}}$$

In this study, GT_EE refers to the energy expenditure (EE) per second calculated based on the indirect calorimetry formula, as defined in Equation (1). The proposed reward function is based on the absolute error between the predicted energy expenditure (Pred_EE) and the ground truth energy expenditure (GT_EE), and is computed according to Equation (10). The objective of the agent is to make predictions that are as close as possible to the actual values. Accordingly, the reward function is designed to guide the learning process toward minimizing the magnitude of the prediction error.

Although mean squared error (MSE) is commonly used in reinforcement learning reward functions, this study adopts an absolute error-based approach. This is because the prediction values in this context are relatively small, and using MSE may result in insufficient reward sensitivity for effective learning.

In addition, normalization was implemented in the reward function by introducing a denominator. This normalization was applied to compensate for variations in the relative error according to the magnitude of GT_EE, thereby facilitating stable learning. By normalizing the error with respect to the magnitude of GT_EE, the reward function provides consistent learning feedback across a wide range of energy expenditure values. This contributes to a more stable learning process.

The reward is always negative and approaches zero as the prediction error decreases. This structure encourages the agent to revise its policy in the direction of reducing the prediction error, providing a clear learning objective. This reward function is particularly well-suited for the task of second-by-second energy expenditure prediction, and the agent progressively improves its estimation accuracy by adjusting its action through iterative learning.

### 3.3. Proposed EEE

The DQN model determines the activity intensity coefficient based on the policy established by considering the state information. The determined activity intensity coefficient is then transferred to EEE for predicted EE calculation. EEE computes the predicted EE by utilizing the activity intensity coefficient (a) received from AF-RL. The equation for calculating the predicted EE based on the activity intensity coefficient is derived from Equations (3) and (5). According to Equation (3), EE is calculated by multiplying the RMR by the MET index. Similarly, Equation (5) states that EE can be calculated by adding the activity energy expenditure (AEE) to RMR. Therefore, Equation (11) is derived as follows.

(11) $$Pred\;{EE}_{sec}={RMR}_{sec\;}\times (1+a)$$

Here, $Pred{EE}_{sec}$ represents the predicted EE per second and ${RMR}_{sec\;}$denotes the RMR per second, calculated using the Mifflin formula (Equations (6) and (7)) in units of seconds. Additionally, a is the activity intensity coefficient (a) predicted by AF-RL. This equation can be explained as follows: The activity intensity coefficient is added to RMR per second, which corresponds to the 1 MET state. The value a represents the MET index excluding the default state of 1, and multiplying this value by RMR yields AEE. Real-time EE prediction is achievable using this equation.

The predicted EE, calculated according to Equation (11), is transferred to the AF-RL Environment and used to compute the reward for the action performed by the Agent, as defined in Equation (10). By iteratively executing this process, the Agent gradually enhances the accuracy of its EE predictions. The RTEE model proposed in this study predicts personalized EE in real time based on reinforcement learning (RL). In summary, AF-RL learns the optimal activity intensity coefficient by considering personal physiological data and heart rate measurements using the DQN-based RL model. The predicted a is applied to the newly defined Equation (11) in EEE to compute the predicted EE. Through this iterative process, the RL agent receives rewards and gradually enhances EE prediction accuracy. The RTEE model enables real-time EE prediction through this structured framework. Furthermore, it is designed for smartwatch implementation, allowing EE prediction using only basic personal information and heart rate data.

## 4. Experiment

### 4.1. Dataset

In this study, we trained and validated the RTEE model, which performs energy expenditure (EE) prediction using the Wearable Energy Expenditure Estimation (WEEE) dataset [40]. The WEEE dataset is a multidevice, multimodal dataset specifically collected for EE estimation research. It contains synchronized biometric information, including heart rate, oxygen consumption (VO_2_), and accelerometer readings, collected from a total of 17 participants using various wearable devices.

VO_2_ was measured using the VO_2_ Master Analyzer, a portable face mask-based system for respiratory gas analysis. The VO_2_ data collected by this device were used as the ground truth (GT) for training and evaluating the RTEE model. Although the VO_2_ Master Analyzer has been validated in previous studies for its accuracy under various exercise conditions, the credibility of the dataset is further supported by multiple research efforts that have utilized the WEEE dataset for EE-related studies [41,42,43].

The WEEE dataset includes second-by-second measurements of oxygen consumption VO_2_ [mL/kg/min] collected using the VO_2_ Master Analyzer. In this study, the VO_2_ [mL/kg/min] data were used together with Equation (1) to generate the ground truth energy expenditure (GT_EE). Since the GT_EE value generated by Equation (1) represents the energy expenditure per minute, it was divided by 60 to convert it to a per-second basis for use as the ground truth data in model training and evaluation.

Table 2 shows the activity status of the participants across different time periods. In the WEEE dataset, 17 participants performed Resting, Cycling, and Running in 10 min intervals, collecting data for a total duration of 30 min. The activity intensity changed every 5 min within each 10 min activity period. During the Resting stage, participants sat for the first 5 min and then stood for the remaining 5 min. In the Cycling stage, participants initially cycled at low intensity (Cycling 1) for 5 min, followed by increased intensity cycling (Cycling 2) for another 5 min. Similarly, in the Running stage, participants engaged in low-intensity running (Running 1) for 5 min, followed by higher-intensity running (Running 2) for the next 5 min. The intensity levels for Cycling and Running varied based on the physical ability of each participant.

Table 3 presents the data items used from the WEEE dataset. First, VO_2_ data, collected using the VO_2_ Master-Analyzer face mask, is utilized to generate ground truth (GT) data. Wrist heart rate, measured by the Empatica E4 device, serves as input data for the model. Additionally, demographic data, including biometric information such as height and weight, is input into the model to derive the activity intensity coefficient and calculate the RMR. Demographic data, representing the physical characteristics of the experiment participants, is also provided. Furthermore, EE data collected from the Apple Watch is used to evaluate and compare its performance with the RTEE model proposed in this study.

### 4.2. Preprocessing

In this experiment, out of the 17 participants in the WEEE dataset, data from 15 participants were used for training, while data from 2 participants were used for testing.

Table 4 presents the average values of the training and test datasets. The dataset was divided into train and test sets, and the average biometric information of each group of participants is displayed. N represents the number of participants used for each corresponding item. The values in the table represent the average measurements for each biometric variable, while the values in square brackets indicate the minimum and maximum values. To ensure an unbiased sample selection for accurately evaluating the model’s performance, the test set was selected to include one male and one female from the total of 17 participants.

**Figure 4:**
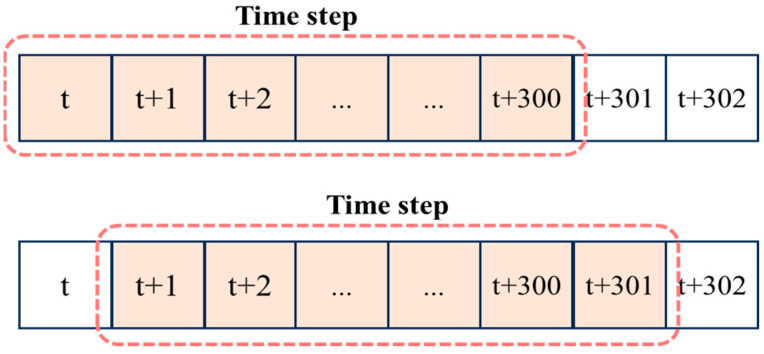
The configuration method of environment.

Figure 4 shows the configuration method of Environment. From the 30 min dataset of a single participant, a total of 300 s of data, spanning from time t to time t + 300, is selected as the Environment. Once learning for a given Environment is completed, the next Environment is defined by shifting the window forward by one second, selecting data from time t + 1 to time t + 301, and the learning process continues. The Environment is determined using this sliding window method.

Table 5 presents the MET values for each activity across all participants. While all participants share the same MET value in the resting state, their exercise intensities vary during Cycling and Running, depending on their physical ability and individual conditions. Consequently, each participant’s activity MET differs. The corresponding MET values provided in the WEEE dataset are used to calculate MET-based EE, following Equation (2).

### 4.3. Experimental Environment

Table 6 summarizes the key hyperparameters used for training the policy network in this study. The Adam optimizer was employed with a learning rate of 1 × $10^{-4}$. A discount factor (γ) of 0.95 was applied to balance immediate and future rewards. The replay buffer size was set to 50,000 to stabilize training through experience replay.

## 5. Result

### 5.1. Performance Comparison by Algorithm

To evaluate the performance of the proposed model, we compared its energy expenditure (EE) prediction results with those obtained from conventional estimation methods, specifically the MET-based method (Equation (2)) and the Keytel equation (Equation (4)), both of which have been widely used in previous studies.

The MET method estimates EE based on a pre-defined MET value and body weight, requiring prior knowledge of the MET index corresponding to the activity type. The Keytel equation estimates EE using heart rate along with personal information such as Gender, weight, and age. Table 7 summarizes the EE estimation formulas presented earlier, along with the required input parameters for each method and the proposed model.

**Figure 5:**
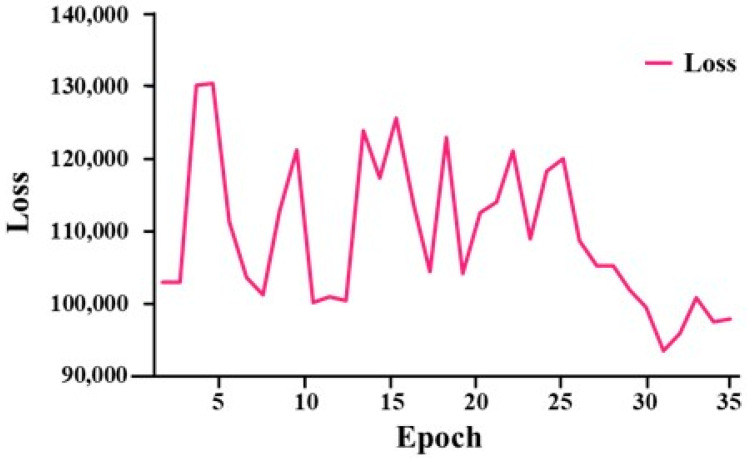
Loss graph per epoch.

In this study, we analyzed the changes in loss values to verify the convergence behavior of the proposed DQN-based model. As shown in Figure 5, the loss exhibited relatively large fluctuations during the early stages of training but gradually decreased over time. Eventually, the loss stabilized within the range of approximately 99,000 to 120,000, indicating that the policy network effectively learned to approximate the Q-values. Training was terminated at 35 epochs, at which point the loss was judged to have sufficiently converged.

The experiment was conducted by training the prediction model using the proposed TREE and then validating it with data from participants who were not included in the training process. The experimental results are visualized in graphs comparing the prediction outcomes of GT EE, Keytel EE, MET EE, and our model. Additionally, the overall error and activity-specific errors for each participant are presented in a table as MAE values. Furthermore, similar to the graph comparing the EE prediction results with those of our model, as provided by the Apple Watch in the WEEE dataset, the MAE errors are also summarized in the table.

**Figure 6:**
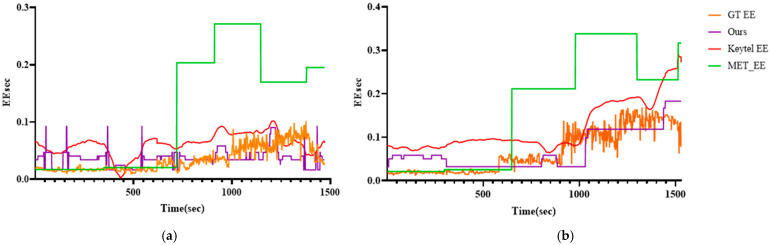
Visualization results of GT EE, our model, Keytel EE, and MET EE: (**a**) P03 participant; (**b**) P09 participant.

Figure 6a shows the prediction results for participant P03, while Figure 6b presents the results for participant P09. For performance verification, the results are shown alongside GT EE, Keytel EE, and MET EE. GT EE represents the EE per second calculated based on Equation (1), while our model (Ours) predicts EE per second using RTEE, as proposed in this paper. Keytel EE is computed using Equation (4), and MET EE is derived from Equation (2). Examining both (a) and (b), we observe that MET EE exhibits the smallest error relative to GT EE during the initial rest period. However, the gap between GT EE and MET EE widens significantly once the activity begins. This indicates that the MET-based EE calculation method is relatively accurate during rest but tends to overestimate EE during activities, leading to a significant reduction in accuracy. Furthermore, Keytel EE consistently overestimates EE throughout the entire duration.

**Figure 7:**
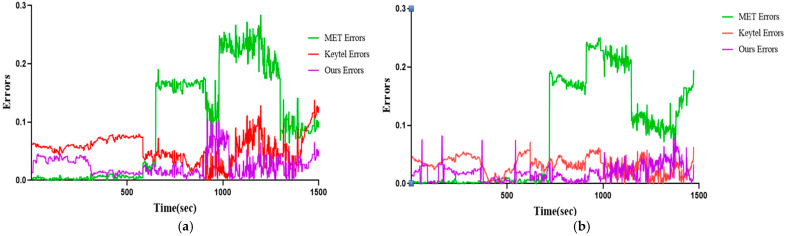
Visualization results of our model Errors, Keytel Errors, and MET Errors: (**a**) P03 participant; (**b**) P09 participant.

In contrast, our model (Ours) demonstrates the smallest error range across all activities. The detailed activity-specific and overall errors are presented below. Figure 7 visualizes the errors of GT EE and each measurement method based on the experimental results in Figure 5. Similar to the prediction results, it can be observed that the MET method exhibits the lowest error during the resting state, while the proposed model achieves the lowest error during active states, particularly at higher exercise intensities.

Table 8 shows the overall and activity-specific MAE errors of EE prediction results for RTEE in participants P03 and P09, along with the MAE errors of Keytel EE and MET EE. The prediction results indicate that the proposed model achieves the lowest prediction error compared to GT EE across the entire measurement period. An analysis of prediction performance by activity segment shows that during the resting state, the MET method produced the lowest error for both participants. However, as they transitioned into the active state, RTEE recorded the lowest prediction error across all activity sections except for the ‘Run2’ section of P03.

In general, while MET EE provides accurate predictions during rest, its error increases significantly during activities. Similarly, Keytel EE consistently overestimates EE across all segments. Therefore, the results demonstrate that the proposed RTEE model most accurately predicts EE across the entire measurement period.

### 5.2. Comparison with Apple Watch

In the previous experiments, we compared the performance of our model (Ours) with the Keytel EE and MET EE calculation methods, which rely on predefined formulas. Additionally, to evaluate how well our model performs against the latest techniques, we compared it with the EE estimates provided by the Apple Watch Series 6, one of the most widely used wearable devices. The EE data from the Apple Watch was obtained from the WEEE dataset.

**Figure 8:**
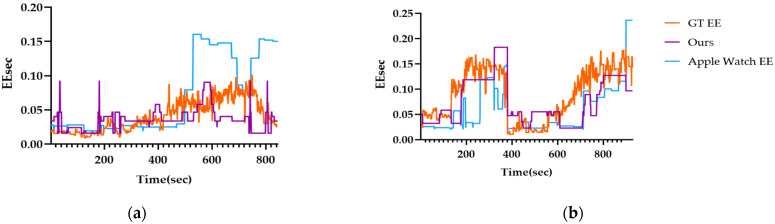
The comparison of measurement results between Apple Watch 6 and Ours EE: (**a**) P03 participant; (**b**) P09 participant.

Figure 8a shows a comparison between GT EE and the predicted EE from our model (Ours) during the activities of participant P03, while Figure 8b compares the EE measured by the Apple Watch with the predicted results. The analysis excludes missing time intervals from the 30 min measurement period.

Examining Figure 8a, we observe that when EE increases, the Apple Watch 6′s predicted EE also rises significantly, leading to substantial prediction errors. In contrast, Figure 8b shows that when EE increases sharply, the Apple Watch 6 does not reflect this increase accurately and instead predicts lower EE values. Overall, these results indicate that our model (Ours) provides more precise EE predictions than the Apple Watch. Below is a table presenting the MAE errors for both the Apple Watch 6 and our model.

**Figure 9:**
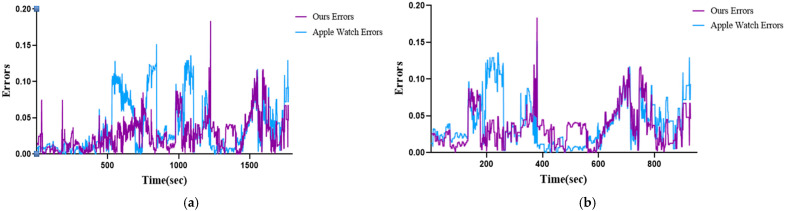
The comparison of measurement results between Apple Watch 6 and Ours Errors: (**a**) P03 participant; (**b**) P09 participant.

Figure 9 visualizes the errors recorded by the Apple Watch and the proposed model compared to the ground truth EE. Overall, the proposed model demonstrated comparable performance to the Apple Watch during lower-intensity activities while showing smaller errors as the exercise intensity increased.

Table 9 shows the performance error for each participant by calculating the MAE error through a comparison of Apple Watch Series 6 EE and our model’s (Ours) predicted EE with GT EE. As shown in the table, both participants (P03 and P09) exhibit lower EE prediction errors with our model than with the Apple Watch 6. Notably, participant P03 achieved a prediction error of just 0.019583, indicating a very low error.

### 5.3. Performance Comparison According to DQN Scenario Length

In RL, performance can vary significantly depending on the scenario length setting. In this study, we conducted two performance tests for prediction accuracy, using scenario lengths of 5 min (300 s) and 1 min (60 s).

**Figure 10:**
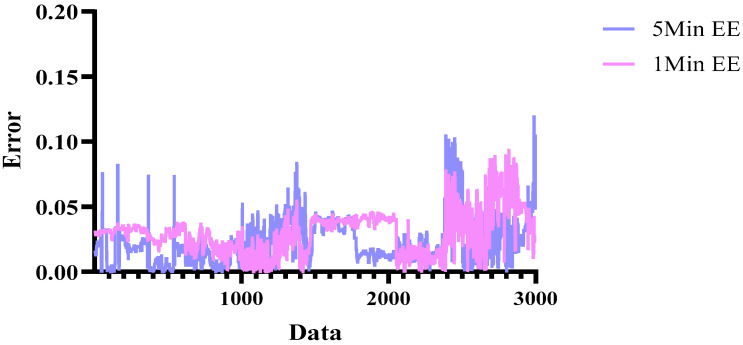
The results of DQN scenario length-based performance evaluation.

Figure 10 shows the EE prediction results from an experiment comparing performance with scenario lengths of 5 min and 1 min. A 5Min EE represents the MAE error across the entire test dataset (participants P03 and P06) when trained with a 5 min scenario length, while 1Min EE represents the MAE error when trained with a 1 min scenario length. The visualization results indicate that while 1Min EE exhibits lower errors in certain areas, 5Min EE demonstrates lower overall errors across the entire dataset. Below is a graph illustrating the MAE errors for both scenario lengths.

Table 10 shows the MAE error of EE prediction based on scenario length. As shown in the table, the prediction error is lower when the scenario length is set to 5 min compared to 1 min.

## 6. Conclusions

In this study, we proposed RTEE, a real-time energy expenditure (EE) prediction model that infers activity intensity from heart rate and applies reinforcement learning. RTEE successfully reflects the Metabolic Equivalent of Task (MET) in real time and mitigates EE overestimation at high intensities. It also leverages the Keytel method to address the variable relationship between heart rate and oxygen consumption, improving prediction accuracy during rest.

RTEE outperformed conventional methods by achieving the lowest average error across various activities, making it particularly suitable for monitoring EE in workers performing diverse tasks rather than single-type exercises. Unlike traditional MET-based approaches, which require activity-specific indices, RTEE estimates intensity coefficients directly from heart rate changes, enhancing real-time usability.

Although the model does not explicitly model the delay in reaching a steady physiological state, it partially captures transition dynamics via heart rate. Future work will focus on modeling these dynamics more precisely and improving accuracy during exercise onset.

We also acknowledge limitations related to the use of RMR (estimated via the Mifflin equation) instead of BMR and dataset dependency. To address variability and approximation errors, our model dynamically adjusts predictions through reinforcement learning. Further work will involve refining BMR–RMR modeling, expanding the dataset, and reducing measurement noise to enhance robustness and generalizability. The model’s performance is subject to the quality of the training data, which may include noise from movement or sensor errors. Future research will aim to enhance reliability by addressing these issues.

## Figures and Tables

**Table 1:** Representative MET index by activity type.

MET	Activity
1.0	Resting energy expenditure (e.g., sitting quietly)
1.5	Office work
4.0	Cycling 10 mph
8.0	Food serving
13.0	Firefighting
16.0	Cycling over 20 mph

**Table 2:** Activity types by time zone.

Time (min)	Task	State
0~5	Sitting	Resting
5~10	Standing	
1~15	Cycling 1	Cycling
15~20	Cycling 2	
20~25	Running 1	Running
25~30	Running 2	

**Table 3:** Sensors and data used in this paper among the WEEE data sets.

Device	Position	Collection Data	Sampling	Unit	Purpose
VO_2_ Master-Analyzer face mask	Mouth	VO_2_	1Hz	mL/kg/min	Ground Truth (GT) Generation
Empatica E4 wristband	Wrist	HR	1Hz	bpm	Activity Intensity Coefficient Prediction
Apple Watch Series 6	Wrist	Energy Expenditure	1Hz	kJ	Result Comparison
Demographic	-	Height, Weight, Age, Gender, MET	-	-	Participant Biometric Information

**Table 4:** The average of training and test data.

	Train (*N* = 15)	Test (*N* = 2)
Age (year)	30.2 (23–41)	26.5 (26–27)
Gender	F, M	F, M
Height	172.2 (160–186)	174.5 (172–175)
Weight	73.6 (53–99)	68.5 (61–76)

**Table 5:** The MET records during the activities of all participants.

Participant	MET_Sit	MET_Stand	MET_Cycle1	MET_Cycle2	MET_Run1	MET_Run2
P01	1	1.2	10	16	4.5	10
P02	1	1.2	10	12	8	14
P03	1	1.2	12	16	10	11.5
P04	1	1.2	10	16	10	15
P05	1	1.2	10	16	8	11.5
P06	1	1.2	10	16	8	11.5
P07	1	1.2	12	16	9	13.5
P08	1	1.2	4	12	8	15
P09	1	1.2	10	16	11	15
P10	1	1.2	16	16	8	11
P11	1	1.2	8	16	8	10
P12	1	1.2	16	16	11.5	18
P13	1	1.2	10	16	8	13.5
P14	1	1.2	6	16	8	11
P15	1	1.2	12	16	15	20
P16	1	1.2	16	16	15	20
P17	1	1.2	16	16	13.5	15.5

**Table 6:** Hyperparameter information.

Hyperparameter	Optimizer	Learning Rate	Discount Factor (γ)	Replay Buffer Size
Value	Adam	$1\;\times \;10^{-4}$	0.95	50,000

**Table 7:** Comparison of EE estimation formulas and parameters.

EEE	Equation	Parameters
MET	$EE(kcal/min)=\frac{MET\times Weight\times 3.5}{200}$	MET, Weight
Keytel	$EE\left(kJ/min\right)=Gender\times (-55.0969+0.6309\times {HR}_{act}+0.1988\times Weight+0.2017\times Age+\left(1-Gender\right)\times \left(-20.4022+0.4472\times {HR}_{act}-0.1263\times Weight+0.074\times Age\right)$	$\mathrm{Gender},{\;HR}_{act}$, Weight, Age
Ours	$EE(kcal/s)={RMR}_{sec\;}\times (1+a)$	${RMR}_{sec\;}$ , a

**Table 8:** The MAE error results of Ours, Keytel EE, and MET EE for P03 and P09 participant data.

	Method	Total Result	Sitting	Standing	Cycle1	Cycle2	Run1	Run2
P03	Ours	0.018459	0.01506	0.011537	0.00759	0.019817	0.025787	0.027091
	Keytel [14]	0.031632	0.041076	0.028461	0.034328	0.033545	0.027634	0.021515
	MET [8]	0.085453	0.002217	0.005383	0.173841	0.221814	0.161276	0.150234
P09	Ours	0.026858	0.036723	0.014454	0.024654	0.029505	0.029961	0.072703
	Keytel [14]	0.056053	0.056798	0.066135	0.033132	0.05192	0.070552	0.182088
	MET [8]	0.097793	0.003062	0.009768	0.154185	0.222327	0.091864	0.213386

**Table 9:** The comparison of MAE error results between Apple Watch and Ours.

	P03	P09
Apple Watch (Series 6)	0.034307	0.05702
**Ours**	**0.019583**	**0.03091**

**Table 10:** The results of MAE error comparison for the performance of Apple Watch and Ours.

DQN Scenario	MAE
5Min EE	0.02266
1Min EE	0.02958

## Data Availability

The data presented in this study are openly available in the WEEE dataset repository at https://wearableenergyexpenditure.github.io/ (accessed on 22 May 2025) at 10.1038/s41597-022-01643-5, reference number [40].
